# Development of a Novel Scoring System for Predicting the Risk of Colorectal Neoplasia: A Retrospective Study

**DOI:** 10.1371/journal.pone.0157269

**Published:** 2016-06-10

**Authors:** Tomohiko Ohno, Seiji Adachi, Mitsuru Okuno, Yohei Horibe, Naoe Goto, Midori Iwama, Osamu Yamauchi, Takao Kojima, Koshiro Saito, Takashi Ibuka, Ichiro Yasuda, Hiroshi Araki, Hisataka Moriwaki, Masahito Shimizu

**Affiliations:** 1 Department of Gastroenterology/Internal Medicine, Gihoku Kosei Hospital, Yamagata, 501–2105, Japan; 2 Division for Regional Cancer Control, Gifu University Graduate School of Medicine, Gifu, 501–1194, Japan; 3 Department of Gastroenterology/Internal Medicine, Gifu University Graduate School of Medicine, Gifu, 501–1194, Japan; University Hospital Llandough, UNITED KINGDOM

## Abstract

**Objective:**

The purpose of this study was to develop a novel scoring system to screen subjects who have a high risk for colorectal neoplasia.

**Study Design and Setting:**

We retrospectively analyzed 1061 subjects undergoing total colonoscopy (TCS) for the first time at Gihoku Kosei Hospital. The characteristics and habits of the subjects were analyzed using a multivariate logistic regression analysis. The risk score was established according to each odds ratio of the individual risk factors, and the correlations between the sum of the risk scores and the prevalence of colorectal neoplasia for each individual were evaluated.

**Results:**

Age 45–59 (risk score: 2 points) and ≥60 (3 points), male gender (1 point), and habitual alcohol consumption ≥21g daily (1 point) were extracted as the significant risk factors for colorectal neoplasia. When the risk groups were determined by summing up these risk scores, the prevalence rates of colorectal neoplasia were 8.8% for the low risk group (0–2 points), 30.5% for the low-moderate risk group (3 points), 39.1% for the high-moderate risk group (4 points), and 57.6% for the high risk group (5 points). In comparison with the low risk group, the odds ratio of the low-moderate risk, the high-moderate risk, and the high risk groups were 4.6, 6.7, and 14.1 folds, respectively.

**Conclusion:**

Our scoring system, which linearly correlates with the prevalence rate of colorectal neoplasia, may be an effective tool for screening the subjects who have a high risk for colorectal neoplasia. These subjects, therefore, should be recommended to undergo TCS.

## Introduction

Colorectal cancer (CRC) is a serious healthcare problem worldwide due to its substantial morbidity and mortality [1]. Therefore, the development of effective screening strategies for this malignancy is urgently required. The annual fecal immunochemical test (FIT) is one of the most effective screening methods against CRC because it reduces the odds risk of CRC-related death ranging from 0.19 to 0.54 [2–5]. In Japan, the annual FIT for individuals over 40 years of age has been applied to mass screening for CRC. However, the death rate of CRC patients has increased by two-fold, up to 41.6% in males and 33.6% in females, compared with the data 20 years prior. Therefore, CRC is now the third leading cause of cancer death in males and the first in females in Japan (http://www.mhlw.go.jp/). These findings suggest that annual FIT screening may be an insufficient mass screening strategy for reducing the mortality of CRC.

Because most CRCs evolve from colorectal adenomas, the early detection and timely resection of colorectal adenoma should prevent death due to CRC [6, 7]. The detection rates of the FIT and total colonoscopy (TCS) for CRC were 0.3% and 0.5%, respectively, and there is little difference between both examinations. However, the detection rates of the FIT for advanced (2.4%) and non-advanced (1.1%) colorectal adenoma were significantly less than those of TCS (9.7% and 22.1%, respectively) [8], which suggests that annual FIT screening may be insufficient for detecting precancerous colorectal lesions. On the other hand, TCS is regarded as an effective strategy for screening colorectal polyps and cancer, as well as preventing CRC-related death, which are stated in both the American Cancer Society (ACS) recommendation and EU guidelines [9, 10].

TCS is recognized as the gold standard method for detecting intraluminal colorectal lesions. However, it is still uncertain whether periodic screening with TCS is better than annual FIT screening when comparing their cost performances and effectiveness. For instance, a previous study showed that TCS screening every 10 years was inferior to annual FIT screening in the prevention of death from CRC and this may be associated with differences in the screening interval between TCS and FIT [11]. More frequent screening with TCS may not be readily recommended to all asymptomatic individuals due to the potential risk of complications such as gut tract perforation. Therefore, in order to detect colorectal neoplasia and reduce its related death, it is urgently necessary to develop a new mass screening system with high efficiency, safety, and convenience. In addition, it is also important to clarify which subjects should undergo TCS to screen the development of colorectal neoplasia.

Several studies have revealed the correlation between the increased risk of CRC or advanced adenoma and individual factors, such as age, sex, lifestyle, lifestyle-related diseases, previous CRC history, familial CRC history or current medications [12–24]. In the present study, we investigated which risk factors are statistically critical for the early detection of colorectal neoplasia, which includes CRC and colorectal adenoma. We also attempted to score the risk factors of colorectal neoplasia and elucidate whether the sum of the scores can lead us to identify which patients are at high risk for developing colorectal neoplasia and thus should undergo screening with TCS.

## Methods

### Subjects

This study was a retrospective case series of consecutive patients who underwent TCS at Gihoku Kosei Hospital for the first time from July 2012 to March 2014. A total of 1061 Japanese subjects were enrolled in this study. All TCSs were performed to screen the colorectum of subjects who showed a positive result at a FIT screening or who had various symptoms such as abdominal discomfort, hematochezia, or constipation. All the subjects were recommended by their doctors to undergo a TCS examination since it had been deemed to be a medical necessity. All the TCSs were performed by expert colonoscopists who had experience more than 3000 colonoscopies. Patients with inflammatory bowel diseases, familial adenomatous polyposis or hereditary non-polyposis colorectal cancer were excluded in this study because they are known to be a high risk group for CRC [13, 25]. All subjects provided their written informed consent before enrollment. The study protocol was approved by the ethics committee of Gihoku Kosei Hospital and followed the tenets of the Declaration of Helsinki.

### Development of the risk scoring system for colorectal neoplasia

The subjects were carefully interviewed for life-style related diseases (diabetes mellitus, hypertension, and hyperlipidemia) and life-styles (alcohol consumption and smoking) prior to undergoing TCS. Prescriptions for diabetes mellitus, hypertension, and hyperlipidemia were also carefully confirmed. The correlation between the individual factors (age, body mass index (BMI), gender, complications, habits, and current medications) and the prevalence of colorectal neoplasia (CRC, advanced adenoma and non-advanced adenoma) was evaluated retrospectively by a univariate analysis using the Pearson χ^2^ method. Advanced adenoma was defined as an adenoma measuring ≥10mm in diameter, with villous architecture (>25%), high-grade dysplasia, or intramucosal carcinoma [26]. A multivariate logistic regression analysis was then carried out for the factors associated with colorectal neoplasia in the univariate analysis (p < 0.05), in addition to the factors that have been previously reported to increase the risk of CRC but were not significant in the present study. For each risk factor, we assigned a weight using the respective adjusted odds ratios (ORs) obtained by the logistic regression analysis.

The risk factors, which were significant in the multivariate analysis, were used to develop the risk scoring system for colorectal neoplasia. The total sum points of each risk score were classified into four risk groups: the low risk (LR), low-moderate risk (LMR), high-moderate risk (HMR), and high risk (HR) groups, respectively, according to the prevalence ratio of colorectal neoplasia. The potential risks of colorectal neoplasia in each risk group were then evaluated using the multivariate logistic regression analysis.

### Statistical analysis

The statistical analysis was performed using the JMP^®^ 10 software program (SAS Institute Inc., Cary, NC, USA). The Pearson χ^2^ test was used for categorical data to compare the proportions of each candidate risk factor. Fisher’s exact test was applied to the categorical data when one of the categorical numbers was less than 10. A multivariate logistic regression analysis was used to analyze the risk factors for colorectal neoplasia. Values of *P* < 0.05 were considered to be statistically significant.

## Results

### Characteristics of the patients

The baseline characteristics of the 1061 patients {629 men (59%) and 432 women (41%); median age, 66.1 years} are shown in Table 1. The median BMI was 22.3 kg/m^2^ (range, 12.3–35.4 kg/m^2^). As for their complications, 158 cases had diabetes mellitus (14.9%), 417 cases had hypertension (39.3%), and 264 cases had hyperlipidemia (24.8%). As for their habits, the median alcohol consumption was 8.4 g/day (range, 0–240 g/day) and 225 cases were smokers (21.2%). The prevalence of the current medications for lifestyle-related diseases for the subjects is also listed in Table 1.

**Table 1:** Characteristics of patients (n = 1061).

**Age (years); mean (range)**	66.1	(17–96)
**Body mass index (kg/m**^**2**^**); mean (range)**	22.3	(12.3–35.4)
**Gender (%)**		
Male	629	(59)
Female	432	(41)
**Life-style related diseases (%)**		
Diabetes mellitus	158	(14.9)
Hypertension	417	(39.3)
Hyperlipidemia	264	(24.8)
**Life-styles**		
Alcohol consumption (g/day); mean (range)	8.4	(0–240)
Smoking (%)	225	(21.2)
**Current medications (%)**		
**Antidiabetic**		
Sulfonylurea	40	(3.8)
Biguanides	25	(2.4)
Thiazolidines	21	(2.0)
Alpha-glucosidase inhibitors	37	(3.5)
Glinides	2	(0.2)
Dipeptidyl peptidase-4 inhibitors	68	(6.4)
Insulin	11	(1.0)
**Hypotensive**		
Angiotensin-converting enzyme inhibitors	42	(4.0)
Angiotensin-2 receptor blockers	212	(20)
Calcium channel blockers	248	(23.4)
Beta blockers	37	(3.5)
Alpha blockers	61	(5.7)
**Lipid-lowering**		
Statins	168	(15.8)
Fibrates	27	(2.5)
**Antiplatelet**		
Aspirin	80	(7.5)
Thienopyridines	43	(4.0)
Phosphodiesterase-3 inhibitors	14	(1.3)
**Anticoagulant**		
Warfarin potassium	27	(2.5)
**Nonsteroidal anti-inflammatory drugs**	129	(12.2)

### Univariate and multivariate predictors of colorectal neoplasia

**Fig 1:**
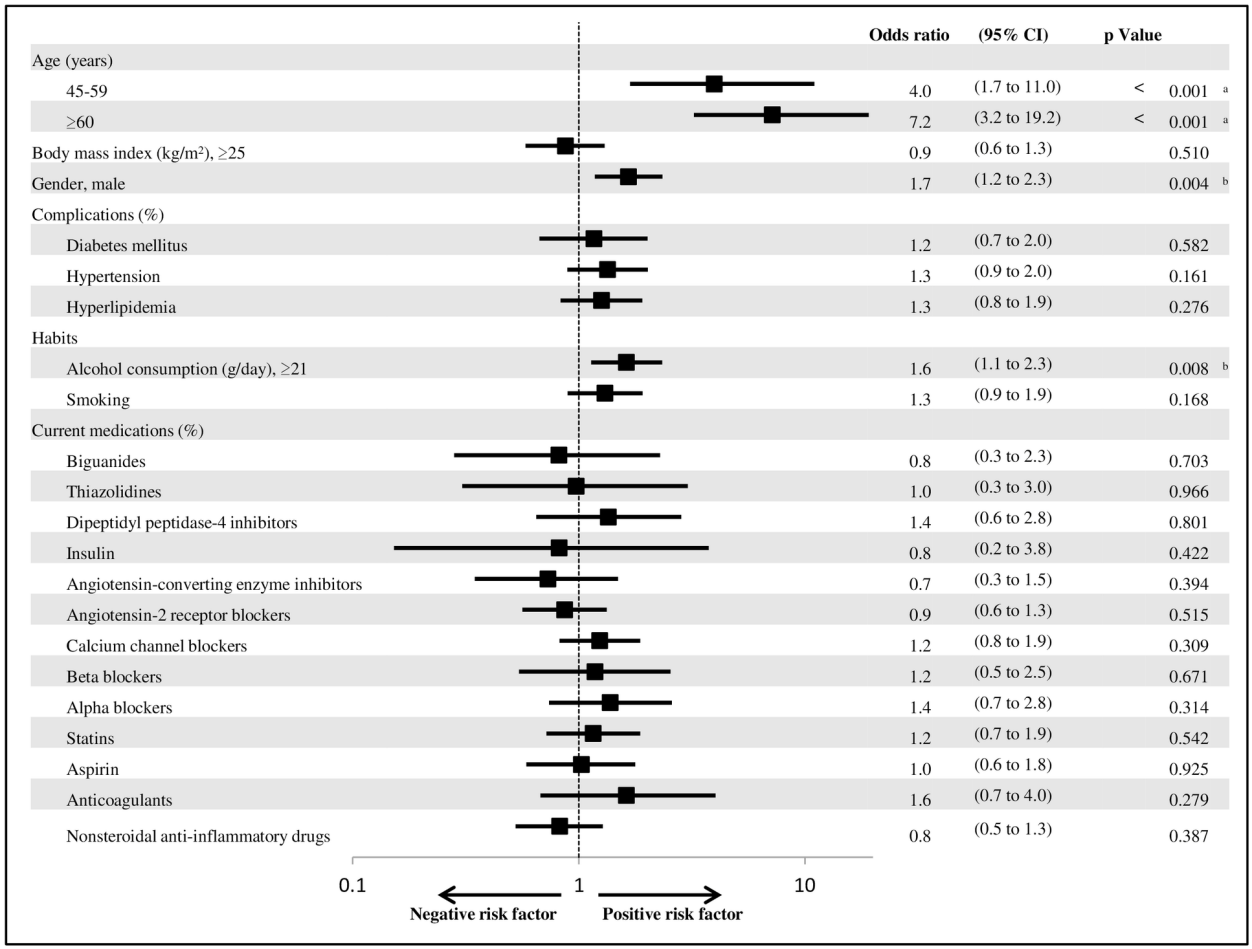
Multivariate predictors of colorectal neoplasia analyzed using a multiple logistic regression analysis. The plot shows the odds ratios (black squares), and 95% CIs (horizontal lines). P-values show the interaction between the prevalence of colorectal neoplasia and any subgroup variable. a. Significant risk factor of colorectal neoplasia by the Pearson χ^2^ test (P < 0.001). b. Significant risk factor of colorectal neoplasia by the Pearson χ^2^ test (P < 0.01).

Univariate and multivariate analyses were performed to identify the predictors of colorectal neoplasia. Among the investigated factors, age 45–59 (p < 0.001) and ≥60 (p < 0.001), male gender (p < 0.001), complications with diabetes mellitus (p = 0.014) and hypertension (p < 0.001), alcohol consumption ≥ 21 g/day (p < 0.001), smoking (p = 0.002), taking of thiazolidines (p = 0.031), dipeptidyl peptidase-4 inhibitor (p = 0.031), angiotensin-2 receptor blockers (ARBs) (p = 0.017), calcium channel blockers (p < 0.001), beta blockers (p = 0.033), alpha blockers (p = 0.001), statins (p = 0.031) and anticoagulants (p = 0.007) were significant risk factors for colorectal neoplasia in the univariate analysis (Table 2). Among these variables, age 45–59 {OR = 4.0, 95% confidence interval (CI) = 1.7–11.0, p < 0.001} and ≥60 (OR = 7.2, 95% CI = 3.2–19.2, p < 0.001), male gender (OR = 1.7, 95% CI = 1.2–2.3, p = 0.004), and alcohol consumption ≥21 g/day (OR = 1.6, 95% CI = 1.1–2.3, p = 0.008) were independent factors associated with an increased risk of colorectal neoplasia according to a multivariate analysis (Fig 1). The cut-off of alcohol consumption was established by the National Health Promotion Movement in the 21st century (Health Japan 21), which set the standard amount of daily alcohol consumption at <21 g/day based on the previous report [27].

**Table 2:** Univariate predictors of colorectal neoplasia.

	Odds ratio	(95% CI)	p Value	
**Age (years)**				
45–59	4.7	(2.3 to 11.1)	<0.001	***
≥60	7.8	(4.0 to 17.6)	<0.001	***
**Body mass index (kg/m**^**2**^**), ≥25**	1.0	(0.7 to 1.4)	0.904	
**Gender, male**	1.9	(1.5 to 2.5)	<0.001	***
**Life-style related diseases (%)**				
Diabetes mellitus	1.5	(1.1 to 2.2)	0.014	*
Hypertension	1.7	(1.3 to 2.3)	<0.001	***
Hyperlipidemia	1.3	(1.0 to 1.7)	0.084	
**Life-styles**				
Alcohol consumption (g/day), ≥21	2.4	(1.7 to 3.4)	<0.001	***
Smoking	1.6	(1.2 to 2.1)	0.002	**
**Current medications (%)**				
**Antidiabetic**				
Sulfonylurea	1.7	(0.9 to 3.2)	0.090	
Biguanides	1.7	(0.8 to 3.9)	0.167	
Thiazolidines	2.5	(1.1 to 6.1)	0.031	*
Alpha-glucosidase inhibitors	0.6	(0.3 to 1.3)	0.167	
Glinides	NA		0.122	
Dipeptidyl peptidase-4 inhibitors	1.7	(1.0 to 2.8)	0.031	*
Insulin	1.1	(0.3 to 3.7)	1.00	
**Hypotensive**				
Angiotensin-converting enzyme inhibitors	1.0	(0.5 to 2.0)	0.918	
Angiotensin-2 receptor blockers	1.5	(1.1 to 2.0)	0.017	*
Calcium channel blockers	1.8	(1.3 to 2.4)	<0.001	***
Beta blockers	2.0	(1.0 to 3.9)	0.033	*
Alpha blockers	2.3	(1.4 to 3.9)	0.001	**
**Lipid-lowering**				
Statins	1.4	(1.0 to 2.0)	0.031	*
Fibrates	0.9	(0.4 to 2.1)	0.857	
**Antiplatelet**				
Aspirin	1.1	(0.7 to 1.8)	0.621	
Thienopyridines	0.6	(0.3 to 1.3)	0.188	
Phosphodiesterase-3 inhibitors	2.5	(0.9 to 7.3)	0.080	
**Anticoagulants**				
Warfarin potassium	2.8	(1.3 to 6.1)	0.007	**
**Nonsteroidal anti-inflammatory drugs**	1.1	(0.8 to 1.6)	0.569	

***Significant risk factor of colorectal neoplasia by Pearson's χ2 test (P < 0.001)

**Significant risk factor of colorectal neoplasia by Pearson's χ2 test (P < 0.01)

*Significant risk factor of colorectal neoplasia by Pearson's χ2 test (P < 0.05)

### Classification of the subjects using the scoring system

In order to develop the risk scoring system for colorectal neoplasia, we created a formula using the significant risk factors, such as age, male gender, and alcohol consumption ≥21 g/day, which were confirmed by the multivariate analysis (Fig 1). The points given to each risk factor were as follows: 2 points to age 45–59, 3 points to age ≥60, 1 point to male gender, and 1 point to alcohol consumption ≥21 g/day. In order to set up these points, the square roots of the ORs for age ≥60 years (7.2), age 45–59 years (4.0), male gender (1.7), and alcohol consumption ≥21 g/day (1.6) were returned, and the decimal points were rounded to the nearest unit, as the statistical analysis clearly demonstrated that the obtained point would best predict the risk of colorectal neoplasia (Table 3).

**Table 3:** Risk Score.

Risk factor	Score
**Age (years)**	
≤44	**0**
45–59	**2**
≥60	**3**
**Sex**	
female	**0**
male	**1**
**Alcohol (g/day)**	
<21	**0**
≥21	**1**

The risk score calculated by the total sum of the points for risk factors present in an individual has a range of 0–5 points according to the presence or absence of risk factors. In this analysis, 933 subjects who had complete clinical datasets were enrolled, and the distribution of the number of subjects with colorectal neoplasia for each score was determined. Some data regarding those risk factors could not be obtained from the subjects, as the interview sheet had been filled out voluntarily. Therefore, the subjects with incomplete clinical datasets were excluded from this analysis. Among the subjects, colorectal neoplasia was observed in 318 subjects (34.1%). Since the prevalence rate was relatively low in the subjects with score 0 (3.7%), score 1 (7.1%), and score 2 (11.8%), the subjects whose score was not greater than 2 were classified as one group and the remaining subjects were arbitrarily divided into four risk groups: score 0–2 as the low risk (LR) group; score 3 as the low-moderate risk (LMR) group; score 4 as the high-moderate risk (HMR) group; and score 5 as the high risk (HR) group. Using the scoring system, 137/933 subjects (14.7%) were classified into the LR group, 344/933 subjects (36.9%) into the LMR group, 320/933 subjects (34.3%) into the HMR group, and 132/933 subjects (14.2%) into the HR group. As listed in Table 4, the prevalence of colorectal neoplasia in each group (LR, LMR, HMR, and HR) was 8.8% (12/137), 30.5% (105/344), 39.1% (125/320), and 57.6% (76/132), respectively. The ORs of the LMR, HMR, and HR groups compared to the LR group were 4.6 (95% CI = 2.5–9.1), 6.7 (95% CI = 3.7–13.2), and 14.1 (95% CI = 7.4–29.2), respectively. The detailed breakdown (non-advanced adenoma, advanced adenoma or CRC) of the colorectal neoplasia in each group was also listed in Table 4. The sensitivity and specificity for colorectal neoplasia, advanced adenoma, and CRC are listed in Table 5 when the cut-off lines for screening were set as over LMR, HMR, and HR, respectively.

**Table 4:** Prevalence and risk of colorectal neoplasia in each group.

				Breakdown of the colorectal neoplasia (%)				
Group	Risk score	Total no. of subject	No. of subject with colorectal neoplasia^a^ (%)	Non-advanced adenoma	Advanced adenoma^b^	Colorectal cancer	Odds ratio	(95% CI)
**Low risk (LR)**	**0–2**	137	12 (8.8)	10 (7.3)	1 (0.7)	1 (0.7)	Reference	
**Low-Moderate risk (LMR)**	**3**	344	105 (30.5)	67 (19.5)	18 (5.2)	20 (5.8)	4.6	(2.5–9.1)
**High-Moderate risk (HMR)**	**4**	320	125 (39.1)	83 (25.9)	20 (6.3)	22 (6.9)	6.7	(3.7–13.2)
**High risk (HR)**	**5**	132	76 (57.6)	47 (35.6)	10 (7.6)	19 (14.4)	14.1	(7.4–29.2)
**Total**		933	318 (34.1)	207 (22.2)	49 (5.3)	62 (6.6)		

^a^Colorectal neoplasia was defined as colorectal cancer, advanced adenoma, and non-advanced adenoma.

^b^Advanced adenoma was defined as an adenoma measuring ≥10 mm in diameter, with villous architecture (>25%), high-grade dysplasia, or intramucosal carcinoma.

**Table 5:** Sensitivity and specificity of colorectal cancer, advanced adenoma, and colorectal neoplasia in each cut-off group.

	Colorectal neoplasia^a^		Advanced adenoma^b^		Colorectal Cancer	
Cut-off group	Sensitivity (%)	Specificity (%)	Sensitivity (%)	Specificity (%)	Sensitivity (%)	Specificity (%)
**Over Low-Moderate risk (LMR)**	96.2	20.3	97.7	15.4	98.4	15.6
**Over High-Moderate risk (HMR)**	63.2	59.2	61.2	52.3	66.1	52.8
**Over High risk (HR)**	23.9	90.7	20.4	85.9	30.7	86.7

^a^Colorectal neoplasia was defined as colorectal cancer, advanced adenoma, and non-advanced adenoma.

^b^Advanced adenoma was defined as an adenoma measuring ≥10 mm in diameter, with villous architecture (>25%), high-grade dysplasia, or intramucosal carcinoma.

## Discussion

The prognosis for patients with advanced CRC is still poor and the development effective strategies for screening the high-risk subjects for this malignancy are urgently needed. FIT is widely used as a screening test for CRC, however, the sensitivity of this examination for detecting colorectal neoplasia, especially colorectal adenoma, is insufficient. TCS is the most accurate examination for detecting colorectal neoplasia and, therefore, identifying asymptomatic subjects who should receive TCS is significant for reducing the risk of death by CRC.

The present study demonstrates that three factors, specifically high age, male gender, and alcohol consumption, were independent risk factors for colorectal neoplasia, which is consistent with the results of previous reports [28, 29]. Furthermore, this study clearly demonstrates that the sum of the risk scores provided from the ORs of these risk factors correlates linearly with the prevalence of colorectal neoplasia. Particularly, elderly men (≥60) who habitually drink alcohol to excess are regarded as to be at a very high risk for developing colorectal neoplasia; the risk of colorectal neoplasia in such subjects increases 14.1 fold compared to younger women (45≥) who do not have a drinking habit. We would like to emphasize that this scoring system, which does not require an invasive examination but can be obtained through a medical inquiry only, may be a useful mass screening system for identifying the subjects at a high risk for colorectal neoplasia who should undergo a TCS examination.

Several trials have been pursued to produce a risk scoring system for the prediction of colorectal neoplasia using the combination of the risk factors associated with an increased risk for CRC or adenoma with high-grade dysplasia [29, 30]. Yeoh KG *et al*. [29] recently reported that a scoring system based on age, male gender, family history of CRC, and smoking was useful in selecting asymptomatic Asian subjects who should receive prior TCS to screen colorectal neoplasia. On the other hand, several biases may remain because these previous studies for developing risk scoring systems did not determine the current medications of the subjects [12, 29, 30]. For instance, habitual medications for lifestyle-related diseases, such as angiotensin-converting enzyme inhibitor, ARB, metformin, statin, aspirin, and nonsteroidal anti-inflammatory drugs, are negative risk factors for CRC, whereas insulin is a positive risk factor [18–24]. In this analysis, the current life-style-related medications were included in order to reduce the confounding biases as much as possible, as such biases are inevitable in retrospective studies even though none of them were found to be significant in the multivariate analysis, since they have already been reported to affect the risk of colorectal cancer in previous studies. Therefore, it is likely that our scoring system allowed us to identify more precisely those at a high risk for developing colorectal neoplasia because our study analyzed the current medications for lifestyle-related diseases in addition to the subjects’ characteristics. Although some authors have reported recently how to screen the patients who have had advanced neoplasia (advanced adenoma or cancer) [29, 31], non-advanced adenoma (low-grade adenoma measuring under 10mm in diameter) had been ignored at all. But in actually, colorectal polyp over 6mm in diameter was recommended to resect by colonoscopy because of its potential to already have a malignant component [32, 33] or to be carcinoma in the future [34]. Thus, it is more practical to create new predicting system for colorectal neoplasia including non-advanced adenoma.

However, this study has two limitations. First, the relative importance of non-advanced adenoma varies with the age at examination, unlike advanced adenoma and colorectal cancer. Therefore, further studies should be performed to determine the suitable range of age for this scoring system. Second, this study lacked data on the tumor characteristics. Molecular pathological epidemiology (MPE) is reported to be important when discussing colorectal neoplasia and patient life-styles [35–39]. MPE studies have shown that colorectal neoplasia can be classified into subtypes based on the pathological findings (e.g. smoking is associated with microsatellite instability [MSI]-high tumors, and obesity is associated with non-MSI tumors) [40–42]. Conducting further studies with consideration of these subtypes of colorectal neoplasia based on this MPE concept may help develop more accurate prediction models.

FIT is known to be the simplest and non-invasive approach to CRC screening. However, its consultation ratio remains under 25% in Japan (http://www.mhlw.go.jp/), whereas the ratio exceeds 50% in the United States [43], which may be associated with the increase in the CRC-related mortality in Japan (http://www.mhlw.go.jp/). One of the reasons why the consultation rate of FIT is low is that this examination is time-consuming and laborious. Moreover, the annual FIT screening is an insufficient mass screening for detecting precancerous colorectal lesion, and subsequently reducing the mortality of CRC, as described above. Therefore, we expect that the scoring system proposed in this study can contribute to an increase in the consultation ratio of TCS screening because neither time nor effort are required in this scoring system to identify asymptomatic subjects who are at a high risk for developing colorectal neoplasia. The subjects in the HMR and HR groups are strongly recommended to undergo TCS, based on the sensitivity and specificity in each cut-off group (Table 5). Therefore, performing TCS, especially on subjects in these groups, may be an effective and efficient strategy for detecting colorectal neoplasia, thereby reducing CRC-related death.

It is generally accepted that most CRCs evolve from colorectal adenomas and that the removal of these lesions have been shown to reduce the risk for oncoming CRC [6, 7]. Therefore, an examination system that can screen the development of colorectal adenoma is more preferable when considering the clinical setting. From this viewpoint, our scoring system, which can predict the development of colorectal neoplasia including adenoma, is more advantageous because the conventional mass screening test, including FIT, appears to be lacking in the ability to detect precancerous lesions such as adenoma with low-grade dysplasia [8].

Finally, it should be noted that this scoring system is no more than a speculation of the risk of colorectal neoplasia, whereas FIT screening is a test which is based on objective evidence. Therefore, the combination of this scoring system and FIT screening could complement each test’s respective faults. Furthermore, this scoring system does not require any cost and FIT screening is reasonably priced. Therefore, using this scoring system might be ideal for mass screening to identify those at a high risk for colorectal neoplasia. A prospective study is required to clarify whether the TCS examination for asymptomatic subjects who are determined to be positive by this scoring system actually supports the detection of colorectal neoplasia and, ultimately, whether this scoring system can lead to a reduction in CRC-related deaths.

## Abbreviations

ACS: American Cancer Society
ARB: angiotensin-2 receptor blockers
BMI: body mass index
CI: confidence interval
CRC: colorectal cancer
FIT: fecal immunochemical test
HMR: high-moderate risk
HR: high risk
LMR: low-moderate risk
LR: low risk
MPE: molecular pathological epidemiology
MSI: microsatellite instability
OR: odds ratio
TCS: total colonoscopy
